# Rethinking Lawton’s Environmental Docility Hypothesis as Older People Adopt Gerontechnology Solution

**DOI:** 10.1093/geroni/igaf122.1920

**Published:** 2025-12-31

**Authors:** Stephen Golant

**Affiliations:** University of Florida, Gainesville, Florida, United States

## Abstract

Lawton’s “environmental docility hypothesis” proposed that older people with physical or cognitive limitations have a greater share of their behaviors explained by the qualities of their residential environments. Occupying dwellings, neighborhoods, or communities with restrictive or unsafe physical or social aspects will more likely constrain or endanger their behaviors. The validity of this proposed relationship is troublesome given the large projected worldwide growth of older people with functional limitations threatening their ability to realize both their obligatory and discretionary needs. However, this lecture argues that these controlling environmental relationships will be less apparent as older people—even at advanced ages—actively cope with their limitations by introducing digital and sensor solutions informed by artificial intelligence into their homes. Having more ways to act proactively—their limitations are less predictive of adverse individual outcomes. Lawton would be pleased—his mission was to improve the “good life” of the less able.

